# HULC cooperates with MALAT1 to aggravate liver cancer stem cells growth through telomere repeat-binding factor 2

**DOI:** 10.1038/srep36045

**Published:** 2016-10-26

**Authors:** Mengying Wu, Zhuojia Lin, Xiaonan Li, Xiaoru Xin, Jiahui An, Qidi Zheng, Yuxin Yang, Dongdong Lu

**Affiliations:** 1 School of Life Science and Technology, Tongji University, Shanghai 200092, China

## Abstract

The dysregulation of lncRNAs has increasingly been linked to many human diseases, especially in cancers. Our results demonstrate HULC, MALAT1 and TRF2 are highly expressed in human hepatocellular carcinoma tissues, and HULC plus MALAT1 overexpression drastically promotes the growth of liver cancer stem cells. Mechanistically, both HULC and MALAT1 overexpression enhanced RNA polII, P300, CREPT to load on the promoter region of telomere repeat-binding factor 2(TRF2), triggering the overexpression, phosphorylation and SUMOylation of TRF2. Strikingly, the excessive TRF2 interacts with HULC or MALAT1 to form the complex that loads on the telomeric region, replacing the CST/AAF and recruiting POT1, pPOT1, ExoI, SNM1B, HP1 α. Accordingly, the telomere is greatly protected and enlonged. Furthermore, the excessive HULC plus MALAT1 reduced the methylation of the TERC promoter dependent on TRF2, increasing the TERC expression that causes the increase of interplay between TRET and TERC. Ultimately, the interaction between RFC and PCNA or between CDK2 and CyclinE, the telomerase activity and the microsatellite instability (MSI) are significantly increased in the liver cancer stem cells. Our demonstrations suggest that haploinsufficiency of HULC/MALAT1 plays an important role in malignant growth of liver cancer stem cell.

The development and progression of cancer has been attributed to independent or combined genetic and epigenetic events. There has been remarkable progress in understanding cancer pathogenesis in terms of genetic alterations. However, recent studies have revealed a complex involvement of epigenetic mechanisms in the regulation of gene expression, including methylation, chromatin modification and remodeling, and the diverse activities of non-coding RNAs. Long non-coding RNAs (lncRNAs) are emerging as key molecules in human cancer and cancer stem cells.

Highly upregulated in liver cancer (HULC), an lncRNA, has recently been revealed to be involved in hepatocellular carcinoma development and progression. HULC is the first ncRNA with highly specific up-regulation in hepatocellular carcinoma (HCC), but its functional contributions in this setting have not been determined. HULC has recently been revealed to be involved in hepatocellular carcinoma development and progression^1^,^2^. Silencing of HULC effectively reversed the epithelial-to-mesenchymal transition (EMT) phenotype. HULC may play an important role in the growth and tumorigenesis of human gastric cancer^3^,^4^. Depletion of IGF2BP1 led to an increased HULC half-life and higher steady-state expression levels, indicating a posttranscriptional regulatory mechanism. Importantly, HULC represents the first IGF2BP substrate that is destabilized to initiate the degradation of the lncRNA HULC^5^. HULC was able to heighten the expression levels of CLOCK and its downstream circadian oscillators, such as period circadian clock 1 and cryptochrome circadian clock 1, in hepatoma cells and accelerates hepatocarcinogenesis through disturbing circadian rhythm^2^. HULC functions as an oncogene in hepatoma cells, acting mechanistically by deregulating lipid metabolism^6^. Some studies showed HULC also may serve as a candidate cancer prognostic biomarker^7^.

MALAT1, a highly conserved long noncoding RNA and also known as nuclear-enriched transcript 2 (NEAT2), is deregulated in several types of cancers. MALAT1 modulates the expression of cell cycle genes and is required for G1/S and mitotic progression^8^. MALAT1 was discovered as a prognostic marker for lung cancer metastasis but also has been linked to several other human tumor entities^9^. MALAT1 serves as an oncogene in esophageal squamous cell carcinoma (ESCC), and it regulates ESCC growth by modifying the ATM-CHK2 pathway^10^. knockdown of PRKA kinase anchor protein 9 (AKAP-9) blocked MALAT1-mediated CRC cell proliferation, migration and invasion and MALAT1 may promote CRC tumor development via its target protein AKAP-9^11^. MALAT1 interacts with pre-mRNAs indirectly through protein intermediates^12^. MALAT1 and Menβ, generate a tRNA-like small RNA in addition to the mature lncRNA^13^. NEAT1 and MALAT1 localize to hundreds of genomic sites in human cells, primarily over active genes^14^. MALAT1 is considered the potential significance in mesenchymal stem cells from myeloma patients by directly interacting with Sp1 and LTBP3 promoter to increase expression of LTBP3 gene^15^. MALAT1 interacts with serine/arginine (SR) splicing factors and influences the distribution of these and other splicing factors in nuclear speckle domains^16^. Although the long MALAT1 transcript localizes to nuclear speckles, the small RNA is found exclusively in the cytoplasm^17^. JMJD1A bound to the MALAT1 gene promoter and demethylated histone H3K9 at the MALAT1 gene promoter^18^.

Telomere protection involves the insertion of the 3′ overhang facilitated by telomere repeat-binding factor 2 (TRF2) into telomeric DNA, forming t-loops. Cellular and organismal ageing are intertwined through the effects of the interaction between TRF2 and lamin A/C on chromosome structure^19^. Shelterin protein TRF2 recruits RTEL1 to telomeres in S phase, which is required to prevent catastrophic t-loop processing by structure-specific nucleases. TRF2 in the recruitment of RTEL1 to facilitate t-loop disassembly at telomeres in S phase^20^. Various types of resolvase activities are kept in check by the basic N-terminal domain of TRF2 in order to favor an accurate repair of the stalled forks that occur during telomere replication^21^. TATA-box-binding protein (TBP)-related factor 2 (TRF2) activates TATA-less core promoters that are dependent on a TCT or downstream core promoter element (DPE) motif^22^. Following TRF2 depletion, the levels of the long noncoding RNA TERRA increase and LSD1, which binds TERRA, is recruited to telomeres^23^. The TRF2-dependent remodeling of telomeres into t-loop structures, which sequester the ends of chromosomes, can explain why NHEJ and the ATM signaling pathway are repressed when TRF2 is present^24^. CSB is required for maintaining the homeostatic level of TERRA, telomere length and integrity^25^. Removal of shelterin through conditional deletion of TRF1 and TRF2 reveals the telomere end-protection problem in nonhomologous end-joining (NHEJ) deficient cells^26^. The p53-dependent ubiquitylation and proteasomal degradation of TRF2 are attributed to the E3 ligase activity of Siah1^27^.

In this study, we indicate that HULC, MALAT1 and TRF2 are highly expressed in hepatocellular carcinoma tissues, and present a positive correlation. Furthermore, HULC, MALAT1 overexpression promotes the growth of liver cancer stem cells *in vitro* and *in vivo*. The demonstration that haploinsufficiency of the LncRNA HULC/MALAT1plays an important role in hepatocarcinogenesis.

## Results

### Upregulated lncRNA MALAT1/HULC were positively associated with the TRF2 expression in human liver cancer tissues

**Figure 1:**
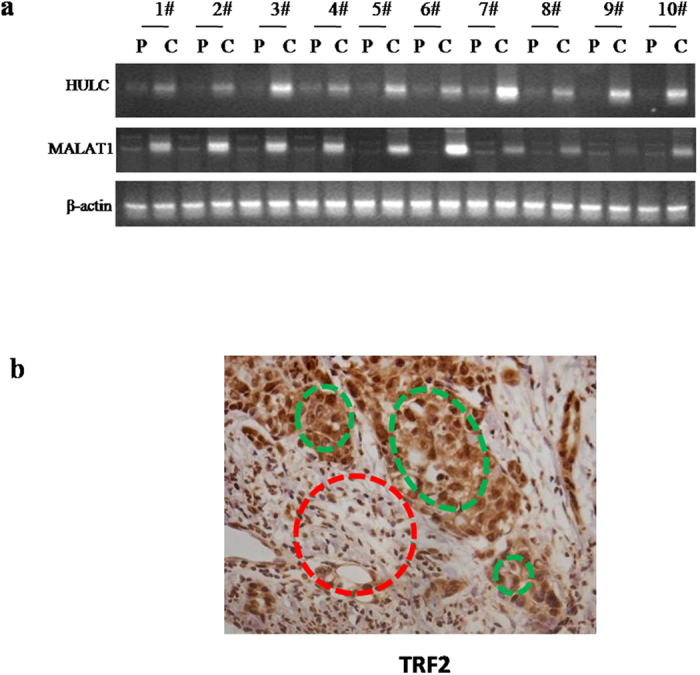
HULC, MALAT1 and TRF2 expression in human liver cancer tissues (**a**). The RT-PCR analysis with HULC and MALAT1 mRNA primer in liver cancer tissue (*C*) and its paracancerous liver tissues (*P*) respectively (indicated in upper). β-actin as internal control. (**b**) The representative analytic results of anti-TRF2 immunohistochemistry staining of formalin-fixed, paraffin-embedded human liver cancer tissues (indicated with green Dotted circles) and their paired adjacent noncancerous tissues (indicated with Dotted red circles) from the same patient. Expression of TRF2 were observed at the cellular level. (DAB stainning, original magnification ×100).

To examine the relationship between long noncoding RNA MALAT1/HULC and TRF2 in human primary liver cancer. We first detected the MALAT1/HULC mRNA in 10 cases of human hepatocarocinoma tissues and their paired adjacent noncancerous tissues from the same patient by RT-PCR. The results showed that the MALAT1/HULC mRNA level was significantly higher in human hepatocarocinoma tissues than their paired adjacent noncancerous tissues, and the upregulation expression rate added up to 100% (n = 10, *t*-test, P < 0.01) (Fig. 1a). Next, we performed immunohistochemistry staining for TRF2 in formalin-fixed, paraffin-embedded 60 case of human hepatocarocinoma tissues and their paired adjacent noncancerous tissues. The results showed the expression of TRF2 was significantly reduced in hepatocarocinoma tissues compared to their paired adjacent noncancerous tissues, and the downregulation expression rate added up to 98.12% (n = 60, *t*-test, P < 0.01) (Fig. 1b). Together, these observations suggest MALAT1, HULC and TRF2 were overexpressed in liver cancer tissue, and there was positively correlation among the expression of MALAT1, HULC and TRF2 in human primary liver cancer.

### MALAT1 plus HULC promotes liver cancer stem cell proliferation

**Figure 2:**
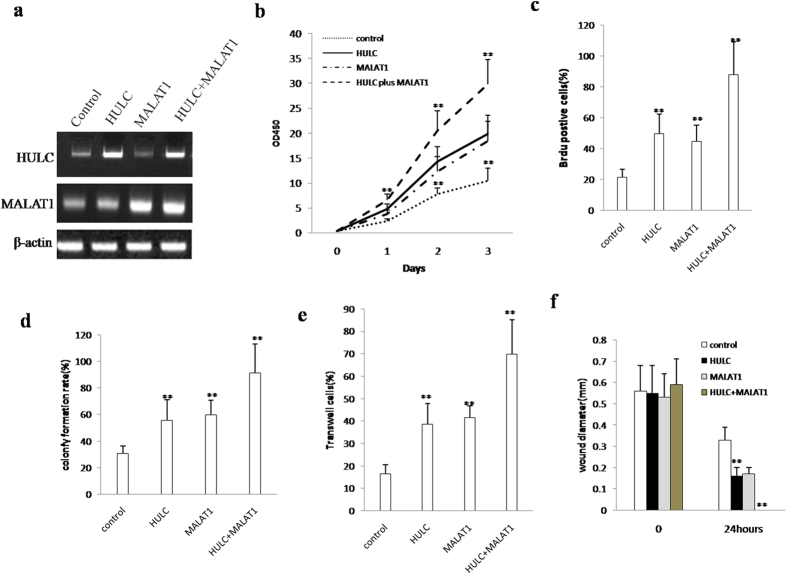
MALAT1 combined with HULC accelerlates liver cancer stem cell growth *in vitro.* (**a**) The RT-PCR analysis of MALAT1 and HULC in stable liver cancer stem cell lines transfected with pCMV6-A-GFP, pCMV6-A-GFP-HULC, pCMV6-A-GFP-MALAT1, pCMV6-A-GFP-HULC plus pCMV6-A-GFP-MALAT1 β-actin as internal control. (**b**) Cells growth assay using CCK8. Each value was presented as mean ± standard error of the mean (SEM). (**c**) S phase cells assay using BrdU. Each value was presented as mean ± standard error of the mean (SEM). (**d**) Cells soft agar colony formation assay. Each value was presented as mean ± standard error of the mean (SEM). (**e**) Cells Transwell assay. Each value was presented as mean ± standard error of the mean (SEM)). (**f**) Wound healing assay. Each value was presented as mean ± standard error of the mean (SEM).

To address the issue whether the MALAT1 combined with HULC influences on liver cancer stem cells malignant proliferation, we established the stable liver cancer stem lines transfected with pCMV6-A-GFP, pCMV6-A-GFP-HULC, pCMV6-A-GFP-MALAT1, pCMV6-A-GFP-HULC plus pCMV6-A-GFP-MALAT1. respectively. As shown in Fig. 2a, HULC was significantly overexpressed in pCMV6-A-GFP-HULC, pCMV6-A-GFP-HULC plus pCMV6-A-GFP-MALAT1 transfected liver cancer stem cells compared the control, and MALAT1 were significantly overexpressed in pCMV6-A-GFP-MALAT1, pCMV6-A-GFP-HULC plus pCMV6-A-GFP-MALAT1 transfected liver cancer stem cell. Next, we detected these cells proliferation *in vitro*. As shown in Fig. 2b, HULC overexpression, MALAT1 overexpression, HULC overexpression plus MALAT1 overexpression promoted liver cancer stem cell cell proliferation compared to control (*t*-test, P < 0.01). Notably, HULC overexpression plus MALAT1 overexpression resulted in the greater extent of promotion. Furthermore, we detected the S phase cells by BrdU staining in HULC or MALAT1 overexpressed liver cancer stem cells. The BrdU positive rate added up to 49.7%, 44.7%, 87.8% in HULC, MALAT1, HULC plus MALAT1 overexpressed liver cancer stem cell, as well as the BrdU positive rate was 21.6% in control (*t*-test, P < 0.01). Especially, the BrdU positive rate was the most highest in HULC plus MALAT1 group (Fig. 2c). Then we performed soft-agar colony-formation efficiency assay in these liver cancer stem cells. The soft-agar colony-formation rates were added up to 55.7%, 59.9%, 91.5% in HULC, MALAT1, HULC plus MALAT1 overexpressed liver cancer stem cells, as well as the soft-agar colony-formation rate was 30.8% in control (*t*-test, P < 0.01). Especially, the soft-agar colony-formation rate was the most highest in HULC plus MALAT1 group (Fig. 2d). The transwell cells positive rates were added up to 38.5%, 41.5%, 69.8% in HULC, MALAT1, HULC plus MALAT1 overexpressed liver cancer stem cells, as well as the transwell cells positive rate was 16.5% in control (*t*-test, P < 0.01). Especially, the transwell cell positive rate was the most highest in HULC plus MALAT1 group (Fig. 2e). The wound diameter was 0.56 mm, 0.55 mm, 0.53 mm, 0.59 mm in control, HULC, MALAT1, HULC plus MALAT1 overexpressed liver cancer stem cells at 0 hours respectively (*t*-test, P < 0.01), as well as the wound diameter was 0.33 mm, 0.16 mm, 0.17 mm, 0 mm in control, HULC, MALAT1, HULC plus MALAT1 overexpressed liver cancer stem cells at 24 hours respectively (*t*-test, P < 0.01) (Fig. 2f). Together, these results suggest that HULC and MALAT1 accelerates the liver cancer stem cells proliferation. HULC plus MALAT1 showed a strong function in the liver cancer stem cell growth.

### MALAT1 combined with HULC accelerlates liver cancer stem cell growth *in vivo*

**Figure 3:**
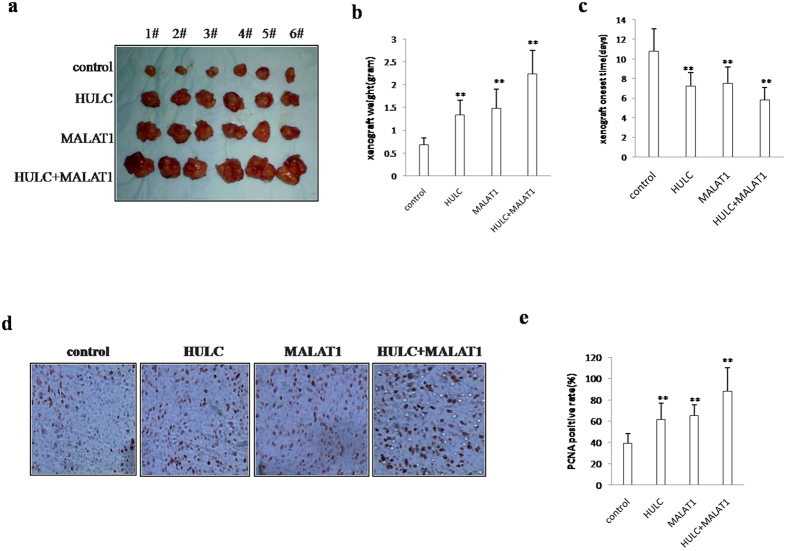
MALAT1 combined with HULC promotes liver cancer stem cell growth *in vivo.* (**a**) The mice were stratified and the tumors were recovered. The photography of xenograft tumors in the four groups (indicated in left). (**b**) The wet weight of each tumor was determined for each mouse. Each value was presented as mean ± standard error of the mean (SEM). (**c**) The Xenograft appearance time (days). Each value was presented as mean ± standard error of the mean (SEM). (**d**,**a**) A portion of each tumor was fixed in 4% paraformaldehyde and embedded in paraffin for histological hematoxylin-eosin (HE) staining (*upper*) and anti-PCNA immunostainning (*lower*). (original magnification × 100). (**b**) PCNA positive cells analysis. Each value was presented as mean ± standard error of the mean (SEM).

To validate whether MALAT1 combined with HULC promotes liver cancer stem cell growth *in vivo*, the stable liver cancer stem cells lines with altered expression of HULC or MALAT1 were injected subcutaneously into Balb/C (severe combined immunodeficiency) mice. As shown in Fig. 3a,b, when HULC was overexpressed, the xenograft tumor weight increased approximately two folds when compared to the corresponding control group (1.34 grams versus 0.68 grams, *t*-test, P < 0.01). When MALAT1 was overexpressed, the xenograft tumor weight increased approximately two folds when compared to the corresponding control group (1.48 grams versus 0.68 grams, *t*-test, P < 0.01). On the other hand, when both HULC and MALAT1 were overexpressed, the average xenograft tumor weight increased to approximately 3.5 folds of the control weight (2.23 grams versus 0.68, *t*-test, P < 0.01). HULC and/or MALAT1 overexpression resulted in early xenograft tumor formation compared to the control group (7.2days, 7.5days, 5.8 days versus 10.8days, respectively *t*-test, P < 0.01) (Fig. 3c). Pathological picture (HE stain) of xenograft tumor showed that tumor tissue possessed more poor-differentiation cells and less moderately or well-differentiation cells in HULC and/or MALAT1 overexpression group than that of control group, suggesting that HULC and/or MALAT1 overexpression enhanced the xenografts tumor malignant grade (Fig. 3c *upper*). The proliferation index (calculated as percentage of PCNA-positive cells) was significantly higher in HULC and/or MALAT1 overexpressed tumors compared to the vector control (88.4%, 61.7%, 65.2% versus 38.9%, respectively, *t*-test, P < 0.01) (Fig. 3 c*lower*&f). Collectively, these findings demonstrate that HULC and/or MALAT1 enhances liver cancer stem cells’ progression *in vivo*.

### MALAT1 or/and HULC enhances TRF2 expression, phosphorylation and sumolyiation

**Figure 4:**
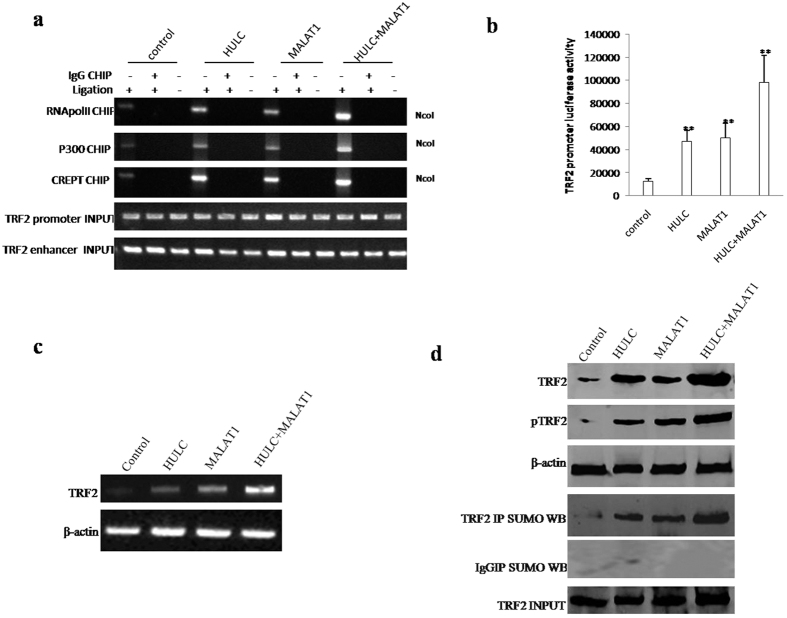
MALAT1 combined with HULC enhances TRF2 expression and sumoylation. (**a**) Chromosome conformation capture (3C)-chromatin immunoprecipitation (ChIP) with anti-P300, anti-RNA polII, anti-CREPT in stable liver cancer stem cell lines transfected with pCMV6-A-GFP, pCMV6-A-GFP-HULC, pCMV6-A-GFP-MALAT1, pCMV6-A-GFP-HULC plus pCMV6-A-GFP-MALAT1. The chromatin is cross-linked, digested with restriction enzymes, and ligated under conditions that favor intramolecular ligation. Immediately after ligation, the chromatin is immunoprecipitated using an antibody (anti-P300, anti-RNA polII) against the protein of interest. Thereafter, the cross-links are reversed, and the DNA is purified further. The PCR anlysis is applied for detecting TRF2 promoter-enhancer coupling product using TRF2 promoter and enhancer primers. The TRF2 promoter and enhancer as INPUT. (**b**) The TRF2 promoter luciferase activity assay in stable liver cancer stem cell lines transfected with pCMV6-A-GFP, pCMV6-A-GFP-HULC, pCMV6-A-GFP-MALAT1, pCMV6-A-GFP-HULC plus pCMV6-A-GFP-MALAT1. (**c**) RT-PCR analysis of TRF2 mRNA. β-actin as internal control. (**d**) Western blotting with anti-TRF2, anti-pTRF2, β-actin as internal control. (*upper*) and anti-TRF2 Co-Immunoprecipitation (IP) followed by Western blotting with anti-SUMO in stable liver cancer stem cell lines transfected with pCMV6-A-GFP, pCMV6-A-GFP-HULC, pCMV6-A-GFP-MALAT1, pCMV6-A-GFP-HULC plus pCMV6-A-GFP-MALAT1. IgG IP as negative control. INPUT refers to Western blotting with anti-TRF2(*lower*).

To confirm whether MALAT1 combined with HULC to enhance TRF2 outcome and its modification, we performed Chromosome conformation capture (3C)-chromatin immunoprecipitation (ChIP), RT-PCR, Western blotting promoter luciferase activity assay and Co**-**Immunoprecipitation (IP). As shown in Fig. 4a, MALAT1 and HULC promotes the TRF2 promoter-enhancer looping formation and enhanced the P300, RNApolII, CREPT entering in the looping. Intriguingly, MALAT1 combined with HULC resulted in the greater efficiency. The luciferase activity assay showed that MALAT1 and HULC promotes the TRF2 promoter luciferase activity. Especifically, MALAT1 combined with HULC resulted in the greater effeciency (Fig. 4b). RT-PCR results showed that MALAT1 and HULC enhanced the TRF2 transcription. Morever, MALAT1 combined with HULC resulted in the greater action (Fig. 4c). Western blotting and Co**-**Immunoprecipitation (IP) results showed that MALAT1 and HULC enhanced the TRF2 expression, its phosphorylation and sumoylation. Especifically, MALAT1 combined with HULC resulted in the greater efficiency (Fig. 4d). Together, MALAT1 combined with HULC to enhance TRF2 expression, phosphorylation, and sumoylation.

### MALAT1 and/or HULC prolongs telomere length

**Figure 5:**
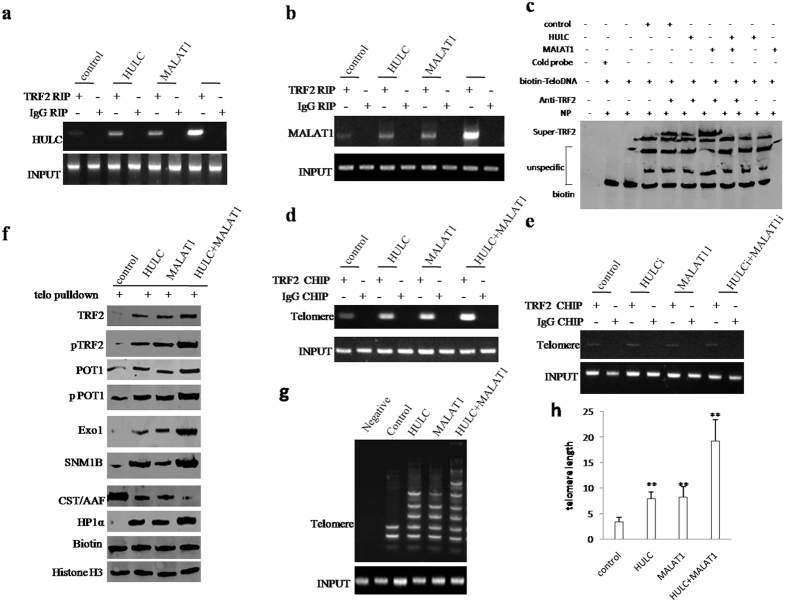
MALAT1 combined with HULC promotes the loading of TRF2 onto telomere that prolongs telomere length. (**a**) RNA Immunoprecipitation (RIP) with anti-TRF2 followed by RT-PCR with HULC mRNA primers in stable liver cancer stem cell lines transfected with pCMV6-A-GFP, pCMV6-A-GFP-HULC, pCMV6-A-GFP-MALAT1, pCMV6-A-GFP-HULC plus pCMV6-A-GFP-MALAT1. IgG RIP as negative control. HULC mRNA as INPUT. (**b**) RNA Immunoprecipitation (RIP) with anti-TRA2 followed by RT-PCR with MALAT1 mRNA primers. IgG RIP as negative control. MALAT1 mRNA as INPUT. (**c**) Super-EMSA (gel-shift) with biotin- telomere DNA probe and anti-TRF2 antibody. The intensity of the band was examined by Western blotting with anti-Bioton. (**d**) Chromatin Immunoprecipitation (CHIP) with anti-TRF2 followed by PCR with telomere DNA primers. IgG CHIP as negative control in stable liver cancer stem cell lines transfected with pCMV6-A-GFP, pCMV6-A-GFP-HULC, pCMV6-A-GFP-MALAT1, pCMV6-A-GFP-HULC plus pCMV6-A-GFP-MALAT1. IgG CHIP as negative control Telomere DNA as INPUT. anti-Bioton. (**e**) Chromatin Immunoprecipitation (CHIP) with anti-TRF2 followed by PCR with telomere DNA primers. IgG CHIP as negative control in stable liver cancer stem cell lines transfected with pGFP-V-RS, pGFP-V-RS–HULC, pGFP-V-RS–MALAT1, pGFP-V-RS-HULC plus pGFP-V-RS-MALAT1. IgG CHIP as negative control Telomere DNA as INPUT. (**f**) Telomere DNA probe Pulldown with anti- TRF2, pTRF2, -POT1, pPOT1, Exo1, SNM1B, CST/AAF, HP1α in stable liver cancer stem cell lines transfected with pCMV6-A-GFP, pCMV6-A-GFP-HULC, pCMV6-A-GFP-MALAT1, pCMV6-A-GFP-HULC plus pCMV6-A-GFP-MALAT1. Histone as internal control. Biotin as INPUT. (**g**) The PCR detection of telomere repeat sequence in stable liver cancer stem cell lines transfected with pCMV6-A-GFP, pCMV6-A-GFP-HULC, pCMV6-A-GFP-MALAT1, pCMV6-A-GFP-HULC plus pCMV6-A-GFP-MALAT1 (**h**). The real-time PCR detection of telomere length in stable liver cancer stem cell lines transfected with pCMV6-A-GFP, pCMV6-A-GFP-HULC, pCMV6-A-GFP-MALAT1, pCMV6-A-GFP-HULC plus pCMV6-A-GFP-MALAT1. Each value was presented as mean ± standard error of the mean (SEM).

Given that MALAT1 combined with HULC to enhance TRF2 expression, phosphorylation, and sumoylation, we had to consider whether MALAT1 combined with HULC altered telomere length. As shown in Fig. 5a, RNA Immunoprecipitation (RIP) with anti-TRF2 showed that MALAT1 and HULC promotes the interaction between HULC and TRF2. Intriguingly, MALAT1 combined with HULC resulted in the greater effeciency in stable liver cancer stem cell lines transfected with pCMV6-A-GFP, pCMV6-A-GFP-HULC, pCMV6-A-GFP-MALAT1, pCMV6-A-GFP-HULC plus pCMV6-A-GFP-MALAT1. As shown in Fig. 5b, RNA Immunoprecipitation (RIP) with anti-TRF2 showed that MALAT1 and HULC promotes the interaction between MALAT1 and TRF2. Intriguingly, MALAT1 combined with HULC resulted in the greater effeciency in stable liver cancer stem cell lines transfected pCMV6-A-GFP, pCMV6-A-GFP-HULC, pCMV6-A-GFP-MALAT1, pCMV6-A-GFP-HULC plus pCMV6-A-GFP-MALAT1. Super-EMSA (gel-shift) with biotin- telomere DNA probe and anti-TRF2 antibody showed that MALAT1 and HULC promotes the interaction between Telomere DNA and TRF2. Intriguingly, MALAT1 combined with HULC resulted in the greater efficiency in stable liver cancer stem cell lines transfected with pCMV6-A-GFP, pCMV6-A-GFP-HULC, pCMV6-A-GFP-MALAT1, pCMV6-A-GFP-HULC plus pCMV6-A-GFP-MALAT1(Fig. 5c). Chromatin Immunoprecipitation (CHIP) with anti-TRF2 showed that MALAT1 and HULC promotes the interaction between telomere DNA and TRF2. Intriguingly, MALAT1 combined with HULC resulted in the greater effeciency in stable liver cancer stem cell lines transfected with pCMV6-A-GFP, pCMV6-A-GFP-HULC, pCMV6-A-GFP-MALAT1, pCMV6-A-GFP-HULC plus pCMV6-A-GFP-MALAT1(Fig. 5d). Notably, this increased interaction between TRF2 and telomere DNA was abrogated when HULC or MALAT1 were knocked down (Fig. 5e). DNA pulldown results showed that MALAT1 and HULC promotes the interaction between telomere DNA and TRF2, pTRF2,-POT1, pPOT1, Exo1, SNM1B, HP1α and inhibited the interaction between telomere DNA and CST/AAF. Intriguingly, MALAT1 combined with HULC resulted in the greater efficiency in stable liver cancer stem cell lines transfected with pCMV6-A-GFP, pCMV6-A-GFP-HULC, pCMV6-A-GFP-MALAT1, pCMV6-A-GFP-HULC plus pCMV6-A-GFP-MALAT1 (Fig. 5f). The regular PCR and real-time PCR detection of telomere repeat sequence showed MALAT1 and HULC increased the telomere length. Intriguingly, MALAT1 combined with HULC resulted in the greater effeciency in stable liver cancer stem cell lines transfected with pCMV6-A-GFP, pCMV6-A-GFP-HULC, pCMV6-A-GFP-MALAT1, pCMV6-A-GFP-HULC plus pCMV6-A-GFP-MALAT1(Fig. 5g,h). Collectively, MALAT1 combined with HULC to prolong telomere length.

### MALAT1 combined with HULC enhanced telomerase activity

**Figure 6:**
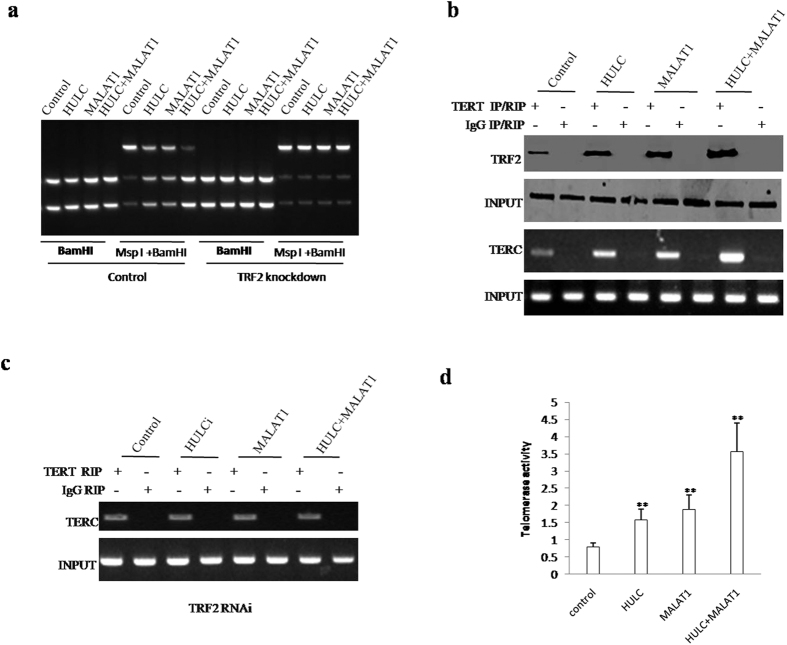
MALAT1 combined with HULC enhanced telomerase activity. (**a**) TERC promoter methylation analysis by MspI plus BamHI digestion in liver cancer stem cell lines transfected with pCMV6-A-GFP, pCMV6-A-GFP-HULC, pCMV6-A-GFP-MALAT1, pCMV6-A-GFP-HULC plus pCMV6-A-GFP-MALAT1 (**b**) (*upper*) Co-Immunoprecipitation (CO-IP) with anti-TERT followed by Western blotting with anti-TRF2. IgG IP as negative control. Western blotting with anti-TERT as INPUT. (*lower*) RNA Immunoprecipitation (RIP) with anti-TERT followed by RT-PCR with TERC mRNA primers. IgG RIP as negative control. TERC mRNA as INPUT. (**c**) RNA Immunoprecipitation (RIP) with anti-TERT followed by RT-PCR with TERC mRNA primers in stable liver cancer stem cell lines transfected with pGFP-V-RS, pGFP-V-RS-HULC, pGFP-V-RS–MALAT1, pGFP-V-RS-HULC plus pGFP-V-RS-MALAT1. IgG RIP as negative control. TERC mRNA as INPUT. (**d**) The telomerase activity was measured by using Quantitative Telomerase Detection. Each value was presented as mean ± standard error of the mean (SEM).

To address whether MALAT1 combined with HULC enhanced telomerase activity, we first analyse the TERC promoter methylation. As shown in Fig. 6a, that MALAT1 and HULC inhibited the TERC promoter methylation. Intriguingly, MALAT1 combined with HULC resulted in the greater action in stable liver cancer stem cell lines transfected pCMV6-A-GFP, pCMV6-A-GFP-HULC, pCMV6-A-GFP-MALAT1, pCMV6-A-GFP-HULC plus pCMV6-A-GFP-MALAT1. Notably, this decreased TERC promoter methylation was abrogated when TRF2 were knocked down (Fig. 6b), suggesting that TRF2 was necessary for the MALAT1-HULC action. Co-Immunoprecipitation with anti-TERT results showed that MALAT1 and HULCpromoted the interaction between TERT and TRF2. Intriguingly, MALAT1 combined with HULC resulted in the greater effeciency in stable liver cancer stem cell lines transfected pCMV6-A-GFP, pCMV6-A-GFP-HULC, pCMV6-A-GFP-MALAT1, pCMV6-A-GFP-HULC plus pCMV6-A-GFP-MALAT1 (Fig. 6c, *upper*). RNA Immunoprecipitation (RIP) with anti-TERT results showed that MALAT1 and HULC promoted the interaction between TERT and TERC. Intriguingly, MALAT1 combined with HULC resulted in the greater effeciency in stable liver stem stem cell lines transfected pCMV6-A-GFP, pCMV6-A-GFP-HULC, pCMV6-A-GFP-MALAT1, pCMV6-A-GFP-HULC plus pCMV6-A-GFP-MALAT1 (Fig. 6c, *lower*). The telomerase activity was measured by using Quantitative Telomerase Detection and the results showed that MALAT1 and HULC increased the telomerase activity. Intriguingly, MALAT1 combined with HULC resulted in the greater effeciency in stable liver cancer stem cell lines transfected pCMV6-A-GFP, pCMV6-A-GFP-HULC, pCMV6-A-GFP-MALAT1, pCMV6-A-GFP-HULC plus pCMV6-A-GFP-MALAT1 (Fig. 6d). Together, these observations suggest that MALAT1 combined with HULC enhanced telomerase activity.

### MALAT1 combined with HULC increases Microsatellite Instability (MSI) and activates cell cycle related proteins

**Figure 7:**
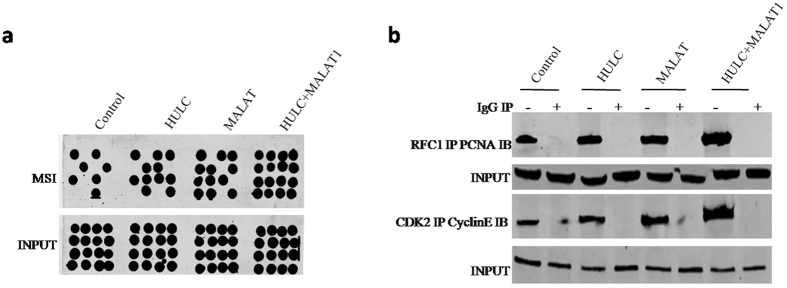
MALAT1 combined with HULC increases Microsatallite Instability (MSI) and cell cycle related protein interplay. (**a**) Microsatallite Instability (MSI) analysis through Dot blot (Slot blot) using various Biotin labling MSI probes (Biotin-MSIs) primers in liver cancer stem cell lines transfected with pCMV6-A-GFP, pCMV6-A-GFP-HULC, pCMV6-A-GFP-MALAT1, pCMV6-A-GFP-HULC plus pCMV6-A-GFP-MALAT1. (**b**) (*upper*) Co-Immunoprecipitation (IP) with anti-RFC1 followed by Western blotting with anti-PCNA. IgG IP as negative control. Western blotting with anti-PCNA as INPUT. (*lower*) Co-Immunoprecipitation (IP) with anti-CDK2 followed by Western blotting with anti-CyclinE. IgG IP as negative control. Western blotting with anti-CyclinE as INPUT.

In stable liver cancer stem cells transfected with pCMV6-A-GFP, pCMV6-A-GFP-HULC, pCMV6-A-GFP-MALAT1, pCMV6-A-GFP-HULC plus pCMV6-A-GFP-MALAT1, we preformed the Microsatallite Instability (MSI) analysis through Dot blot (Slot blot) using various Biotin labling MSI probes (Biotin-MSIs) primers. As shown in Fig. 7a, MALAT1 and HULC increased the Microsatallite Instability (MSI). Intriguingly, MALAT1 combined with HULC resulted in the greater efficiency. Then we preformed the Co-Immunoprecipitation (IP) in these cell lines. As shown in Fig. 7b, MALAT1 and HULC increased the interaction between RFC1 and PCNA, CDK2 and CyclinE. Intriguingly, MALAT1 combined with HULC resulted in the greater effeciency. Together, these observations suggest MALAT1 combined with HULC increases Microsatallite Instability (MSI) and cell cycle related protein interplay.

### TRF2 depletion abrogated the oncogenic function of the MALAT1 and/or HULC

**Figure 8:**
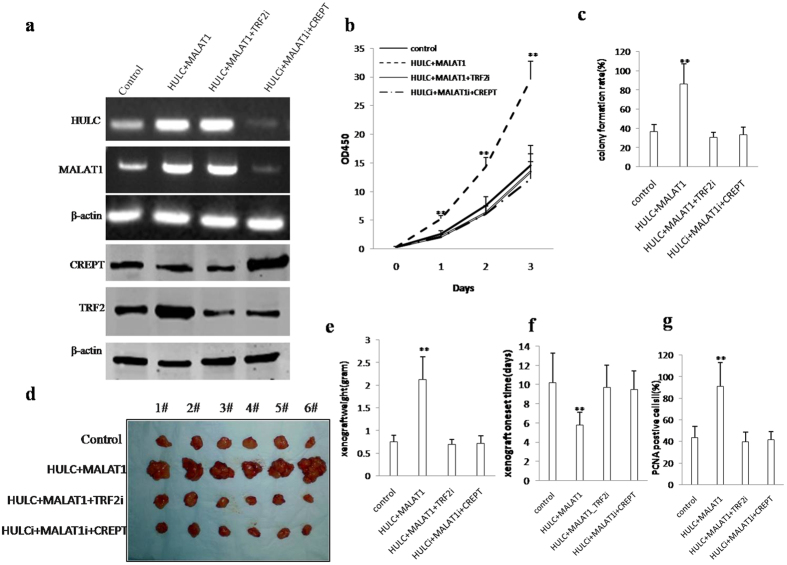
The rescued experiment of carcinogenesis effect of the MALAT1 combined with HULC. TRF2 knockdown abrogated the oncogenic function of the MALAT1 combined with HULC. (**a**) The RT-PCR analysis of MALAT1, HULC (*upper*) and the western blotting analysis with anti-TRF2(*lower*) in stable liver cancer stem cell lines transfected with pCMV6-A-GFP, pCMV6-A-GFP-HULC plus pCMV6-A-GFP-MALAT1, pCMV6-A-GFP-HULC plus pCMV6-A-GFP-MALAT1 plus pcDNA3.1-TRF2, pGFP-V-RS-GFP-HULC plus pGFP-V-RS–MALAT1 plus pcDNA-CREPT. β -actin as internal control. (**b**) Cells growth assay using CCK8. Each value was presented as mean ± standard error of the mean (SEM). (**c**) Cells soft agar colony formation assay. Each value was presented as mean ± standard error of the mean (SEM). (**d**) *In vivo* test in stable liver cancer stem cell lines transfected with pCMV6-A-GFP, pCMV6-A-GFP-HULC plus pCMV6-A-GFP-MALAT1, pCMV6-A-GFP-HULC plus pCMV6-A-GFP-MALAT1 plus pcDNA3.1-TRF2, pGFP-V-RS-GFP-HULC plus pGFP-V-RS–MALAT1 plus pcDNA-CREPT. The mice were stratified and the tumors were recovered. The photography of xerograft tumor in the four groups (indicated in left). (**e**) The wet weight of each tumor was determined for each mouse. Each value was presented as mean ± standard error of the mean (SEM). (**f**) The Xenograft appearance time. Each value was presented as mean ± standard error of the mean (SEM). (**g**) PCNA expression in the Xenografts. Each value was presented as mean ± standard error of the mean (SEM).

To explore whether the oncogenic function of the MALAT1 and/or HULC is dependent on TRF2, we preformed the rescued experiment in liver cancer stem cell line. We constructed the stable liver cell stem cells transfected with pCMV6-A-GFP, pCMV6-A-GFP-HULC plus pCMV6-A-GFP-MALAT1, pCMV6-A-GFP-HULC plus pCMV6-A-GFP-MALAT1 plus pGFP-V-RS-TRF2, pGFP-V-RS-GFP-HULC plus pGFP-V-RS–MALAT1 plus pcDNA-CREPT respectively. RT-PCR and Western blotting results showed that MALAT1, HULC, TRF2, CREPT were successfully overexpressed or knocked down in these cell lines (Fig. 8a). Cell growth assay results showed that MALAT1 combined with HULC promotes liver cancer stem cells proliferation *in vitro.* However, this action was fully abrogated in liver cancer stem cell lines transfected with pCMV6-A-GFP-HULC plus pCMV6-A-GFP-MALAT1 plus pFGP-V-RS—TRF2 or pGFP-V-RS-GFP-HULC plus pGFP-V-RS –MALAT1 plus pcDNA-CREPT (Fig. 8b). Cell colony formation capicity assay results showed that MALAT1 combined with HULC increased cells colony formation capacity (86.23% vs 36.7%, P < 0.01). However, this action was fully abrogated in liver cancer stem cell lines transfected with pCMV6-A-GFP-HULC plus pCMV6-A-GFP-MALAT1 plus pFGP-V-RS—TRF2 or pGFP-V-RS-GFP-HULC plus pGFP-V-RS –MALAT1 plus pcDNA-CREPT (30.56%, 33.24% vs 36.7%, respectively, *t*-test, P < 0.01) (Fig. 8c). In tumorigenesis test *in vivo*, our results showed that MALAT1 combined with HULC increased xenograft tumors weight (2.12 gram vs 0.75 gram, P < 0.01). However, this action of MALAT1 combined with HULC was abrogated in liver cancer stem cell lines transfected with pCMV6-A-GFP-HULC plus pCMV6-A-GFP-MALAT1 plus pFGP-V-RS-TRF2 or pGFP-V-RS-GFP-HULC plus pGFP-V-RS –MALAT1 plus pcDNA-CREPT (0.69 gram, 0.72 gram vs 0.75 gram, respectively, *t*-test, P < 0.01) (Fig. 8d,e). MALAT1 combined with HULC decreased xenograft tumors appearance time (5.8 days vs 10.2 days, *t*-test, P < 0.01). However, this action was fully abolished in liver cancer stem cell lines transfected with pCMV6-A-GFP-HULC plus pCMV6-A-GFP-MALAT1 plus pFGP-V-RS—TRF2 or pGFP-V-RS-GFP-HULC plus pGFP-V-RS –MALAT1 plus pcDNA-CREPT (9.7days, 9.5 days vs 10.2 days, respectively, *t*-test, P < 0.01) (Fig. 8f). MALAT1 combined with HULC increased PCNA positive rate (91.2% vs 43.7%, *t*-test, P < 0.01). However, this action of MALAT1 combined with HULC was abrogated in liver cancer stem cell lines transfected with pCMV6-A-GFP-HULC plus pCMV6-A-GFP-MALAT1 plus pFGP-V-RS—TRF2 or pGFP-V-RS-GFP-HULC plus pGFP-V-RS–MALAT1 plus pcDNA-CREPT (39.5%, 41.5% vs 43.7%, respectively, P > 0.05) (Fig. 8g). Strikingly, although HULC plus MALAT1 enhances TRF2 transcriptional activity via CREPT, CREPT overexpression could not promote growth of liver cancer stem cell after both HULC and MALAT1 were knocked down in liver cancer stem cells. Collectively, TRF2 knockdown abrogated the oncogenic function of the MALAT1 plus HULC in liver cancer cells.

## Discussion

**Figure 9:**
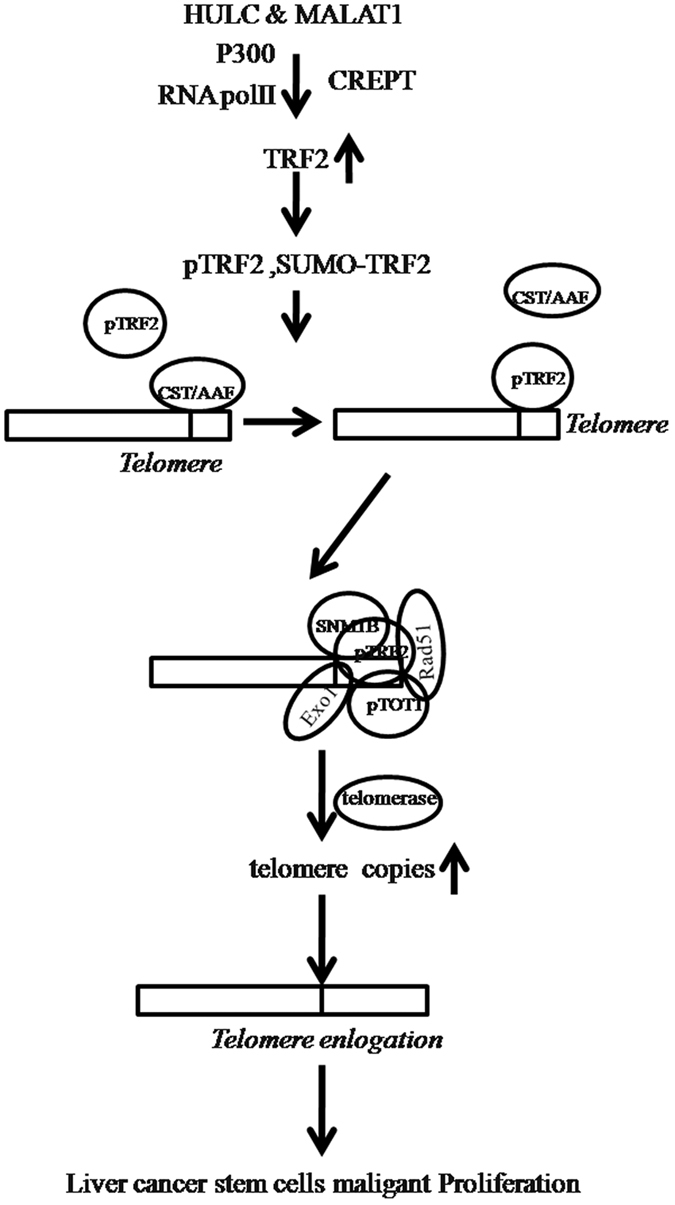
The schematic diagram illustrates a model that The synergetic effect of HULC and MALAT1 promotes liver cancer growth through upregulation of TRF2. Our results show that HULC, MALAT1 and TRF2 are highly expressed in hepatocellular carcinoma tissues, and present a positive correlation. Furthermore, HULC, MALAT1 overexpression promotes the growth of liver cancer stem cells *in vitro* and *in vivo*. Mechanistically, HULC, MALAT1 overexpression resulted in more RNApolII, P300, CREPT to load onto the promoter region of TRF2, enhancing the TRF2 expression at the level of transcription and its phosphorylation and SUMOylation. At the same time, the increased TRF2 binds to HULC, MALAT1 into complex that loads to the telomeric region of the chromosome, replacing the CST/AAF and the recruiting of POT1, pPOT1, ExoI, SNM1B, HP1 α. Accordingly, the telomere is greatly lengthened. On the other hand, the increased TRF2 reduced the methylation of the TERC promoter, increasing the TERC expression that results in a increase of interaction between TRET and TERC, Thus, activity of telomerase is raised. Ultimately, the microsatallite instability (MSI) is increased, and the interaction between RFC and PCNA or between CDK2 and CyclinE is increased in the liver cancer stem cells, which led to the rapid growth of liver cancer stem cells.

To this data, we clearly identify that HULC, MALAT1 and TRF2 are highly expressed in hepatocellular carcinoma tissues, and present a positive correlation. Furthermore, HULC, MALAT1 overexpression promotes the growth of liver cancer stem cells *in vitro* and *in vivo*. Mechanistically, HULC, MALAT1 overexpression resulted in more RNA polII, P300, CREPT to load onto the promoter region of TRF2, enhancing the TRF2 expression at the level of transcription and its phosphorylation and SUMOylation. At the same time, the increased TRF2 binds to HULC, MALAT1 into complex that loads to the telomeric region of the chromosome, replacing the CST/AAF and the recruiting of POT1, pPOT1, ExoI, SNM1B, HP1 α. Accordingly, the telomere is greatly protected. On the other hand, the increased TRF2 reduced the methylation of the TERC promoter, increasing the TERC expression that results in a increase of interaction between TRET and TERC, Thus, activity of telomerase is raised. Ultimately, the microsatellite instability (MSI) is increased, and the interaction between RFC and PCNA or between CDK2 and CyclinE is increased in the liver cancer cells, which led to the rapid growth of hepatocellular carcinoma cells (Fig. 9). To our knowledge, this is the first report demonstrating HULC cooperates with MALAT1 to aggravate liver cancer stem cells growth through telomere repeat-binding factor 2. It is worth mentioning that HULC plus MALAT1 may play an important role in the occurrence of hepatocellular carcinoma. In this report, we focused mainly on the view how HULC plus MALAT1 functions during liver cancer stem cells malignant growth.

To date, accumulating evidence indicates that HULC and MALAT1 have a strong tumorigenic action and are a bona fide oncogene. HULC has been implicated in the regulation of hepatoma cell proliferation. HULC expression is significantly higher in HCC tumors compared to normal liver tissues. Among the tumor tissues, higher HULC expression is positively associated with Edmondson histological grades or with hepatitis B (HBV) positive status. Moreover, HULC lncRNA is detected with higher frequency in the plasma of HCC patients compared to healthy controls^28^. HULC might function through regulating a tumor suppressor gene p18 located near HULC in the same chromosome and the up-regulated HULC by HBx promotes proliferation of hepatoma cells through suppressing p18^29^. Depletion of HULC resulted in a significant deregulation of several genes involved in liver cancer^30^. HULC expression is not confined to HCC, but also to those colorectal carcinomas that metastasize to the liver^31^. MALAT1 (metastasis-associated lung adenocarcinoma transcript 1) is a predictive marker for metastasis development in lung cancer. Antisense oligonucleotides (ASO) blocking MALAT1 prevent metastasis formation after tumor implantation^32^. Overexpression of MALAT1 confers an oncogenic function in renal cell carcinoma (RCC) that may offer a novel theranostic marker in this disease^33^. silencing MALAT1 inhibits highly invasive subline of brain metastasis lung cancer cell migration and metastasis by inducing epithelial-mesenchymal transition (EMT)^34^. The expression of MALAT1 is upregulated in colorectal cancer (CRC) tissues, and a higher expression level of MALAT1 might serve as a negative prognostic marker in stage II/III CRC patients^35^. The lncRNAs MALAT1 has been reported in association with HCC^36^. In addition, knockdown of MALAT1 significantly inhibited the proliferation and metastasis of the gallbladder carcinoma (GBC) and ERK/MAPK pathway was found to be inactivated in the GBC cell lines after MALAT1 knockdown^37^. The lncRNA MALAT1 is an important mediator of TGF-β-induced EMT and malat1 inhibition may represent a promising therapeutic option for suppressing bladder cancer progression^38^. Our present findings are consistent with some reports. It is worth noting that our findings in this study provide novel evidence for an active role of HULC plus MALAT1 promotion of liver cancer stem cell growth. The involvement of promotion of liver cancer stem cells growth based on HULC plus MALAT1 is supported by results from three parallel sets of experiments: (1) Both MALAT1 and HULC were overexpressed in liver cancer tissue. (2) MALAT1 plus HULC promotes liver cancer stem cell proliferation. (3) MALAT1 combined with HULC accelerlates liver cancer stem cell growth *in vivo.*

In our previous report, we have identified a novel gene *CREPT* (***c**ell-cycle **r**elated and **e**xpression- elevated **p**rotein in **t**umor*) (Genebank: DQ372938 DQ372939). We had demonstrated that CREPT was highly expressed in human tumor tissues and accelerated cell growth and tumorigenesis. In particular, CREPT enhances Cyclin D1 expression through promoting RNAPII binding to both the CyclinD1 promoter and the termination region before the poly-A site^39^. In this report, our findings demonstrates CREPT promote HULC combined MALAT1 to active TRF2. In addition, we also reveal CREPT oncogenic action is fully dependent on HULC and MALAT1.

Strikingly, our observations suggest that TRF2 determines the HULC and/or MALAT1 oncogenic action and TRF2 plays a key role for HULC and MALAT1 tumorigeneic function. TRF2 is essential for telomere capping owing to its roles in suppressing an ATM-dependent DNA damage response (DDR) at chromosome ends and inhibiting end-to-end chromosome fusions^40^. The shelterin protein TIN2 binds to TRF1 and TRF2, improving the telomeric localization of TRF2 and its function^41^. Longer telomeres and TRF2 expression in HCCs are associated with poor patient outcomes^42^. TRF2 specifically interacts with the histone acetyltransferase p300, and that p300 acetylates the lysine residue at position 293 of TRF2^43^. Genomic instability resulting from loss of telomeric repeat-binding factor 2 (TRF2) expression provides biological advantages to the cancer stem cell population^44^. Moreover, TRF2 simultaneously binds TERRA and telomeric duplex or G-quadruplex DNA^45^. Apollo, a nuclease bound to the shelterin subunit TRF2, initiates formation of the 3′ overhang at leading-, but not lagging-end telomeres^46^. TERRA associates with SUV39H1 H3K9 histone methyltransferase, which promotes accumulation of H3K9me3 at damaged telomeres and end-to-end fusions^47^. CUDR promotes liver cancer stem cell growth through upregulating TERT and C-Myc^48^. This assertion is based on several observations in HULC and/ot MALAT1 overexpresed liver cancer stem cells: (1) Upregulated lncRNA MALAT1/HULC were positively associated with the TRF2 expression in human liver cancer tissues. (2) MALAT1 or/and HULC enhances TRF2 expression, phosphorylation and sumolyiation. (3) TRF2 knockdown abrogated the oncogenic function of the MALAT1 and/or HULC.

Importantly, accumulating evidence indicates DNA mismatch repair (MMR) ensures replication fidelity by correcting mismatches generated during DNA replication, and telomerase activity and telomere length decides cell fate. LncRNA HOTAIR promotes human liver cancer stem cell malignant growth through inhibition of DNA mismatch repair (MMR)^49^. Of Significance, our results showed that HULC cooperated with MALAT1 to enhances telomerase activity, elongates telomere length and increased Microsatellite Instability (MSI). This assertion is based on several observations in liver cancer stem cells, (1) MALAT1 and/or HULC prolongs telomere length. (2) MALAT1 combined with HULC enhanced telomerase activity. (3) MALAT1 combined with HULC increases Microsatellite Instability (MSI) and cell cycle related protein interplay.

Our previous findings confirm that several long noncoding RNAs are involved in stem cells malignant transformation and hepatocarcinigenesis. For examples, long noncoding RNA CUDR regulates HULC and β-Catenin to govern human liver stem cell malignant differentiation^50^. SET1A cooperates with CUDR to promote liver cancer growth and hepatocyte-like stem cell malignant transformation epigenetically^51^. miR675 upregulates long noncoding RNA H19 through activating EGR1 in human liver cancer^52^. Furthermore, we should explore the function of HULC combined with MALAT1. For example, what causes strong oncogenic action HULC plus MALAT1? How does HULC cooperates with MALAT1? Does HULC plus MALAT1 regulates a series of molecular events liver stem cells malignant growth? Answering these questions will help understand the mechanism about liver stem cell malignant differentiation. In summary, our present data indicated that HULC combined with MALAT1 promotes liver cancer stem cells malignant progression through altering telomere and MSI, with diagnostic and prognostic implications. These observations provide insight into a novel link between noncoding RNA and hepatocarcinigenesis. The demonstration that haploinsufficiency of the LncRNA HULC/MALAT1 is very important in hepatocarcinogenesis. To underscore the need for new approaches uncover the mechanisms underlying lncRNAHULC/MALAT1-mediated functions in hepatocarcinogenesis *in vivo*.

## Experimental Procedures

### Ethics statement

All methods were carried out in “accordance” with the approved guidelines. All experimental protocols “were approved by” a Tongji university institutional committee. Informed consent was obtained from all subjects. The study was reviewed and approved by the China national institutional animal care and use committee”.

### Patients and tissue samples

Sixty cases of paired liver cancer tissues and their adjacent noncancerous liver tissues used for analysis were obtained from liver cancer patients who had undergone surgery. Informed consents were obtained from all patients. All patients were diagnosed as liver cancer according to histological examination. These results were reviewed independently by at least three pathologists or clinicians.

### Human liver cancer stem cell line (hLCSC) sorting

CD133/CD44/CD24/EpCAM MicroBead Kits were purchased from Miltenyi technic (Boston, USA) and MACS^®^ Technology operation according to and the operation according to the manufacturer.

### Cell Lines and Plasmids

Human liver cancer stem cell line (hLCSC) was maintained in Dulbecco’s modified Eagle medium (Gibco BRL Life Technologies) or Minimum Essential Medium (MEM) (Gibco BRL Life Technologies) supplemented with 10% heat-inactivated (56 °C, 30 minutes) fetal bovine serum (sigma) in a humidified atmosphere of 5% CO_2_ incubator at 37 °C. pCMV6-A-HULC, pCMV6-A-MALAT1, pGFP-V-RS-HULC, pGFP-V-RS-MALAT1, pGFP-V-RS-TRF2 and pGL3-TRF2 promoter were cloned by ourselves.

### Cell transfection and stable cell lines

Cells were transfected with DNA plasmids using transfast transfection reagent lipofectamine^R^ 2000 (Invitrogen) according to manufacturer’s instructions. For screening stable cell lines, forty-eight hours after transfection, cells were plated in the selective medium containing G418(1000–2000 μg/ml, Invitrogen) for the next 4 weeks or so, and the selective media were replaced every 3 days.

### RT-PCR

Total RNA was purified using Trizol (Invitrogen) according to manufacturer’s instructions. cDNA was prepared by using oligonucleotide (dT)_17–18_, random primers, and a SuperScript First-Strand Synthesis System (Invitrogen). PCR analysis was performed under the specical conditions. β-actin was used as an internal control.

### Nuclear Run on assay

Nuclear run-on was performed by supplying biotin-probe to nuclei, and labeled transcripts were bound to streptavidin-coated streptavidin-agarose Resin according to the ref. (53).

### Western Blotting

The logarithmically growing cells were washed twice with ice-cold phosphate-buffered saline (PBS, Hyclone) and lysed in a RIPA lysis buffer. Cells lysates were centrifuged at 12,000 g for 20 minutes at 4 °C after sonication on ice, and the supernatant were separated. After being boiled for 5–10 minutes in the presence of 2-mercaptoethanol, samples containing cells proteins were separated on a 10% sodium dodecyl sulfate-polyacrylamide gel electrophoresis (SDS-PAGE) and transferred onto a nitrocellulose membranes (Invitrogen, Carlsbad, CA, USA). Then blocked in 10% dry milk-TBST (20 mM Tris-HCl [PH 7.6], 127 mM NaCl, 0.1% Tween 20) for 1 h at 37 °C. Following three washes in Tris-HCl pH 7.5 with 0.1% Tween 20, the blots were incubated with 0.2 μg/ml of antibody (appropriate dilution) overnight at 4 °C. Following three washes, membranes were then incubated with secondary antibody for 60 min at 37 °C or 4 °C overnight in TBST. Signals were visualized by ODYSSEY infrared imaging system (LI-COR, Lincoln, Nebraska USA).

### Co-immunoprecipitation (IP)

Cells were lysed in 1 ml of the whole-cell extract buffer A (50 mM pH7.6 Tris-HCl, 150 mM NaCl, 1% NP40, 0.1 mM EDTA, 1.0 mM DTT, 0.2 mM PMSF, 0.1 mM Pepstatine, 0.1 mM Leupeptine, 0.1 mM Aproine). Five-hundred-microliter cell lysates was used in immunoprecipitation with antibody. In brief, protein was pre-cleared with 30 μl protein G/A-plus agarose beads (Santa Cruz, Biotechnology, Inc.CA) for 1 hour at 4 °C and the supernatant was obtained after centrifugation (5,000 rpm) at 4 °C. Precleared homogenates (supernatant) were incubated with 2 μg of antibody and/or normal mouse/rabbit IgG by rotation for 4 hours at 4 °C, and then the immunoprecipitates were incubated with 30 μl protein G/A-plus agarose beads by rotation overnight at 4 °C, and then centrifuged at 5000 rpm for 5 min at 4 °C. The precipitates were washed five times ×10 min with beads wash solution (50 mM pH 7.6 Tris Cl, 150 mM NaCl, 0.1% NP-40, 1 mM EDTA) and then resuspended in 60 μl 2 × SDS-PAGE sample loading buffer to incubate for 5–10 min at 100 °C. Then Western blot was performed with a another related antibody indicated in Western blotting.

### RNA Immunoprecipitation (RIP)

Cells were lysed (15 min, 0 °C) in 100 mM KCl, 5 mM MgCl_2_, 10 mM HEPES [pH 7.0], 0.5% NP40, 1 mM DTT, 100 units/ml RNase OUT (Invitrogen), 400 μM vanadyl-ribonucleoside complex and protease inhibitors (Roche), clarified and stored on at −80 °C. Ribonucleoprotein particle-enriched lysates were incubated with protein A/G-plus agarose beads (Santa Cruz, Biotechnology, Inc. CA) together with specific antibody for 4 hours at 4 °C. Beads were subsequently washed four times with 50 mM Tris-HCl (pH 7.0), 150 mM NaCl, 1 mM MgCl_2_, and 0.05% NP-40, and twice after addition of 1 M Urea. Immunoprecipitates (IPs) were digested with proteinase K (55 °C; 30′) and mRNAs were then isolated and purified for RT-PCR.

### DNA pull down

Cells were lysed by sonication in HKMG buffer (10 mM HEPES, PH7.9, 100 mM KCl, 5 mM MgCl_2_, 100% glycerol, 1 mM DTT, and 0.5% NP40) containing protease inhibitors for the preparation of nuclear exact. Equal amount of cell nuclear extracts were precleared with Streptavidin-agarose Resin (Thermo) for 1 hours, and then were incubated with 1 μg biotinylated double-stranded-oligonucleotides together with 10 μg poly (dI-dC) at 4 °C for 24 hours. DNA-bound proteins were collected with the incubation with streptavidin-agarose Resin for 1 hour with gently shaking to prevent precipitation in solution. Following five times washings of the resin bound complex with 0.5–1.0 ml of binding buffer, the samples were boiled and subjected to SDS-PAGE and Western blotting analysis.

### Super-EMSA (Gel-shift)

Cells were washed and scraped in ice-cold PBS to prepare nuclei for electrophoretic gel mobility shift assay with the use of the gel shift assay system modified according to the manufacturer’s instructions (Promega).

### Chromatin immunoprecipitation (CHIP) assay

Cells were cross-linked with 1% (v/v) formaldehyde (Sigma) for 10 min at room temperature and stopped with 125 mM glycine for 5 min. Crossed-linked cells were washed with phosphate-buffered saline, resuspended in lysis buffer, and sonicated for 8–10 min. Chromatin extracts were diluted 5-fold with dilution buffer, pre-cleared with Protein-A/G-Sepharose beads, and immunoprecipitated with specific antibody on Protein-A/G-Sepharose beads. After washing, elution and de-cross-linking, the ChIP DNA was detected by PCR.

### Chromosome conformation capture (3C) -chromatin immunoprecipitation (ChIP) assay (ChIP-3C/ChIP-Loop assays)

Antibody-specific immunoprecipitated chromatin was obtained as described above for ChIP assays. Chromatin still bound to the antibody-Protein-A/G-Sepharose beads were resuspended in 500 μl of 1.2× restriction enzyme buffer at 37 °C for 1 h. 7.5 μl of 20% SDS was added, the mixture was incubated for 1 h, followed by addition of 50 μl of 20% Triton X-100, and then incubation for an additional 1 h. Samples were then incubated with 400 units of selected restriction enzyme at 37 °C overnight. After digestion, 40 μl of 20% SDS was added to the digested Chromatin, and the mixture was incubated at 65 °C for 10 min. 6.125 ml of 1.15× ligation buffer and 375 μl of 20% Triton X-100 was added, the mixture was incubated at 37 °C for 1 h, and then 2000 units of T4 DNA ligase was added at 16 °C for a 4-h incubation. Samples were then de-cross-linked at 65 °C overnight followed by phenol-chloroform extraction and ethanol precipitation. After purification, the ChIP-3C material was detected for long range interaction with specific primers.

### MSI detection through Dot blot (Slot blot)

Dot blots can only confirm the presence or absence of a biomolecule or biomolecules which can be detected by the DNA probes. Various Biotin labling MSI probes (Biotin-MSIs) added individually to the wells where a vacuum sucks the water (with NaOH and NH_4_ OAc) from underneath the membrane (nitrocellulose) as a dot and then is spotted through circular templates directly. The cells DNA is quantified and equal amounts are aliquoted into tubes. These are denatured (NaOH and 95 °C) and can be hybridized with the membrane to allow for the detection of variation between samples. The signal can be detected by anti-Biotin Western blotting.

### DNA CpG Methylation analysis via Restriction Enzyme Cleavage

First, isolate genomic DNA and then digest 10 μg of DNA overnight with BamH, or BamH in combination with MspI. Reaction volume should be about 50 μl. Inactivate the enzymes by heating. Reduce the sample volume to about 20 μl in the vacuum concentrator. Add required volume of loading buffer and load the samples onto the 1% agarose gel.

### Cells proliferation CCK8 Assay

Cells were synchronized in G0 phase by serum deprivation and then released from growth arrest by reexposure to serum, and then cells were grown in complete medium for assay. The cell proliferation reagent CCK8 is purchased from Roch and the operation according to the manufacturer instruction.

### Soft agar colony formation assay

2 × 10^2^ cells were plated on a 6 well plate containing 0.5% (lower) and 0.35% (upper) double layer soft-agar. The 6 well plates were incubated at 37 °C in humidified incubator for 21 days. The cells were fed 1–2 times per week with cell culture media (DMEM). Soft-agar colonies on the 6 well plates were stained with 0.5 ml of 0.05% Crystal Violet for more than 1 hour and the colonies were counted.

### BrdU staining

80% confluent cells were cultured for 24 hour before treatment with 10 μl BrdU (Roche) for 4 hours. Immunofluorescent staining with an anti-BrdU antibody was performed according to the manufacturer’s instructions (Becton Dickinson). BrdU positive cells from ten random chosen fields of at least three independent samples were counted.

### Xenograft transplantation *in vivo*

Four-weeks male athymic Balb/C mice per group were injected with liver cancer stem cells at the armpit area subcutaneously. The mice were observed over 4 weeks, and then sacrificed to recover the tumors. The wet weight of each tumor was determined for each mouse. A portion of each tumor was fixed in 4% paraformaldehyde and embedded in paraffin for histological hematoxylin-eosin (HE) staining. The use of mice for this work was reviewed and approved by the institutional animal care and use committee in accordance with China national institutes of health guidelines.

### Statistical analysis

The significant differences between mean values obtained from at least three independent experiments. Each value was presented as mean ± standard error of the mean (SEM) unless otherwise noted, with a minimum of three replicates. The results were evaluated by SPSS20.0 statistical soft (SPSS Inc Chicago, IL) and Student’s t-test was used for comparisons, with P < 0.05 considered significant.

## Additional Information

**How to cite this article**: Wu, M. *et al*. HULC cooperates with MALAT1 to aggravate liver cancer stem cells growth through telomere repeat-binding factor 2. *Sci. Rep.* **6**, 36045; doi: 10.1038/srep36045 (2016).

**Publisher’s note:** Springer Nature remains neutral with regard to jurisdictional claims in published maps and institutional affiliations.
